# Integrin and Its Associated Proteins as a Mediator for Mechano-Signal Transduction

**DOI:** 10.3390/biom15020166

**Published:** 2025-01-23

**Authors:** Kazuo Katoh

**Affiliations:** Laboratory of Human Anatomy and Cell Biology, Faculty of Health Sciences, Tsukuba University of Technology, Tsukuba 305-8521, Japan; katoichi@k.tsukuba-tech.ac.jp

**Keywords:** mechano-signal transduction, integrins, extracellular matrix (ECM), mechanical cues

## Abstract

Mechano-signal transduction is a process in which cells perceive extracellular mechanical signals, convert them into intracellular biochemical signals, and produce a response. Integrins are cell surface receptors that sense the extracellular mechanical cues and bind to the extracellular matrix (ECM). This binding induces integrin clustering and activation. Cytoplasmic tails of activated integrins interact and induce cytoskeleton tensions via several adaptor proteins. Integrins monitor extracellular stiffness via cytoskeleton tensions and modulate ECM stiffness via downstream signaling pathways regulating the expression of genes of ECM components. Integrin-mediated mechano-transduction is very crucial for the cell as it regulates the cell physiology both in normal and diseased conditions according to extracellular mechanical cues. It regulates cell proliferation, survival, and migration. Abnormal mechanical cues such as extreme and prolonged mechanical stress result in pathological conditions including fibrosis, cancers, skin, and autoimmune disorders. This paper aims to explore the role of integrins and their associated proteins in mechano-signal transduction. It highlights the integrins and their associated proteins as targets for therapy development. Furthermore, it also presents the challenges to the targeted drug development, which can be drug resistance and cytotoxicity. It is concluded in this paper that research on integrin-mediated mechano-signal transduction and its relationship with cell physiology and pathologies will be an important step towards the development of effective therapies.

## 1. Introduction

Mechano-signal transduction is the process of conversion of extracellular biomechanical signals to intracellular biochemical signals. It is very crucial for many physiological processes of cells. The extracellular mechanical signals serve as mechanical cues to the cell’s activity so that the cell can perform its activity by extracellular mechanical conditions. Mostly mechanical cues include hydrostatic compression, liquid shear stress, tensile stress, stiffness of extracellular matrix (ECM), elasticity of tissues, and extracellular fluid viscosity. Mechano-signal transduction potentially triggers several biological processes, including embryonic development, wound healing, and regeneration [1]. However, prolonged and extreme mechanical stimulation causes pathological conditions. Fibrosis, tumorigenesis, and resistance to cancer immunotherapy are the outcomes of prolonged and extreme mechanical stimulation [2]. Integrins are transmembrane cell surface adhesion receptors. They serve as a key player in most of the mechano-signal transduction pathways [3]. The integrin-mediated mechano-transduction is mostly responsible for tissue differentiation, development, and maintenance of homeostasis [3].

Cells can monitor the extracellular biomechanics via integrins because they can sense the biomechanical changes in ECM [4]. In response to the biomechanical cues or changes in the ECM, they bind with the ligands present in the ECM and are subsequently activated [5]. Integrin transfers this biomechanical signal to the cytoplasm in biochemical form and triggers downstream signaling pathways via focal adhesion (FA) formation. These downstream signaling pathways then produce the ultimate cell response to the biomechanical signal [6]. At FA, integrins coordinate the ECM with the cytoskeleton and reorganize it in tensed form via multiple proteins [7]. These FA proteins are mechanosensitive and collectively serve as a molecular clutch that transfers the cytoskeletal tensions back to the ECM. Thereby, FA proteins modulate the elasticity of both the cell and the ECM via reciprocal transmission of tensions from ECM to cell and from cell to ECM [3].

Integrins and associated proteins mediate mechano-transduction, causing the activation of several downstream signaling pathways that regulate cell proliferation, apoptosis, wound healing, and development [5,7,8,9]. MAPK, RhoA, and PI3K are the predominant pathways that trigger upon integrin-mediated mechano-signal transduction [10,11,12]. FAK and c-Src are two cytoplasmic kinases that activate upon ECM signal reception by extracellular domains of integrins [13]. Upon activation, FAK and c-Src phosphorylate and activate other proteins, which leads to the activation of the MAPK pathway. Similarly, integrin-mediated activated FAK and c-Src stimulate PI3K protein, which triggers the PI3K/AKT pathway, thus promoting cell proliferation, growth, and survival. Talin and kindlins are two of the integrin-associated cytoplasmic proteins that are involved in the activation of the RhoA pathway. The RhoA pathway modulates the cytoskeletal elements of the cell, regulating focal adhesion kinetics, cell migration, and adhesion [2,14]. Integrin-mediated mechano-signal transduction also activates downstream pathways, including YAP/TAZ, JNK, mTOR, and Src family kinase pathways. All of these pathways are interconnected and regulate cell division, growth, and repair/healing [2]. In this way, regulating these pathways, integrin and integrin-associated protein-mediated mechano-signal transduction regulates different physiological processes in the body, both in normal and diseased conditions.

Integrin-mediated mechano-signal transduction is associated with the progression of different diseases as well. When integrins sense the increased extracellular load or extreme stress and trigger the downstream pathways, which lead to pathological conditions [15]. These pathological conditions include cancer, fibrosis, muscle and skeletal deformities, autoimmune and inflammatory disorders, neurodegenerative disorders, different congenital disorders including laminopathies and Marfan syndrome, and wound healing anomalies [16,17,18,19]. All these pathological conditions involve the activation of an intricate network of different signaling pathways that are being modulated upon mechanical signal reception from ECM by integrins. Integrin and associated protein-mediated mechano-signal transduction present different potential targets for therapy against different pathological conditions arising from the malfunction of integrin-mediated signal transduction. It has the potential to develop therapy against cancer and fibrosis by targeting integrin or its associated proteins [20,21,22].

This review paper aims to explore how integrins receive mechanical signals from the extracellular matrix and transmit them to the cytoplasm. This paper also analyzes the role of integrin-associated proteins in integrin-mediated mechano-signal transduction. This paper emphasizes the diverse pathologies that result from mechano-signal transduction. This study also figures out the potential targets in mechano-transduction to develop a therapeutic regime against different disorders. Finally, it sheds light on the challenges and future research on integrin-mediated mechano-signal transduction and potential therapeutic targeting. Thus, this paper presents comprehensive research on the integrin and its associated protein-mediated mechano-transduction along with its biological and clinical significance.

## 2. Mechano-Signal Transduction

The process, in which cells convert the extracellular mechanical signals into intracellular biochemical signals and that biochemical signal triggers a cascade of different signaling pathways, is called mechano-signal transduction. Mechano-signal transduction involves a complex network of signaling pathways, which produce the ultimate cell response [23]. The mechano-signal transduction maintains cell homeostasis by sensing the extracellular environmental changes. It is essential for the development of various tissues, wound healing, stem cell activation, and cell division, differentiation, and death [23,24]. Moreover, mechano-signal transduction enables cells to sense enhanced mechanical pressure and forces, which results in ECM remodeling, like in bones, thereby fulfilling the demands of the mechanical pressure [15]. The cell perceives any abnormal change in the extracellular environment, like extreme mechanical force applied, which results in altered mechano-signal transduction. Consequently, several pathological conditions arise from altered cell proliferation, differentiation, death, and migration [20].

**Figure 1:**
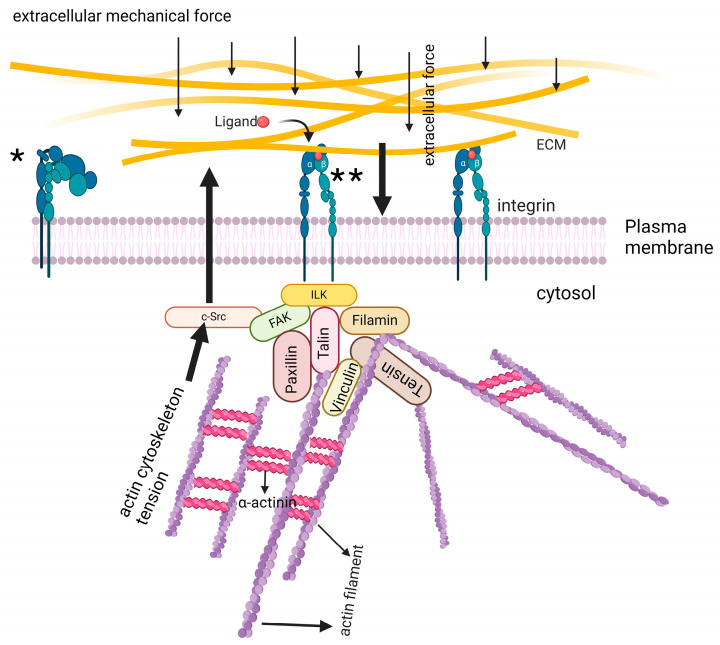
Mechanisms of mechano-signal transduction are summarized. FAK, c-Src, vinculin, talin, F-actin, and other proteins that receive signals from integrin and transfer it to the cytoplasm, thereby triggering a signaling cascade associated with ultimate cell response. * indicates inactivated form of integrins. ** indicates activated form of integrins. Created in https://BioRender.com.

Cell surface receptors, including integrins, serve as sensors for the detection of mechanical changes in the ECM, thereby playing a basic role in mechano-signal transduction. Then cytoplasmic proteins associated with integrins constitute the key role in mechano-signal transduction. These proteins include focal adhesion kinase (FAK), c-Src, vinculin, talin, F-actin, and other proteins that receive signals from integrin and transfer them to the cytoplasm, thereby triggering a signaling cascade associated with the ultimate cell response [3,21]. Thus, integrins and their associated proteins play an integral role in mechano-signal transduction. Mechanisms of mechano-signal transduction are summarized in Figure 1.

### 2.1. Role of Mechano-Signal Transduction

Mechano-transduction is a cellular mechanism that transforms mechanical stimuli, such as stretching, fluid flow, and hydrophobic pressure, into intracellular signal transduction. The intricate chemical pathways that govern mechano-transduction can be convoluted. Nevertheless, certain fundamental physical concepts are adequate to comprehend the mechanisms of mechano-transduction. In well-researched instances of mechano-transduction, particular proteins experience force-induced conformational changes that are linked to variations in catalytic activity or binding affinity for partners. Mechanical pressure induces metabolic alterations that can disseminate through established signal transduction pathways [23]. Conformational alterations induced by mechanical force transpire in numerous major protein complexes and subcellular structures, potentially resulting in both local and global modifications in cellular organization, behavior, proliferation, and differentiation. This review describes cell adhesion to the extracellular matrix (ECM), despite the existence of multiple membrane organization types, such as caveolae [22] and membrane deformation induced by BAR domain–containing proteins [25], which react to mechanical forces at the cell surface.

The mechanisms by which cells detect and transduce exterior forces into intracellular signals have been thoroughly investigated in tissue culture, focusing on single-cell adherence to the extracellular matrix and multicellular structure [26]. As previously noted, a defining feature of numerous proteins that connect external forces from the extracellular matrix to intracellular signaling is their propensity to unfold in reaction to force or tension. Fibronectin is a key constituent of the extracellular matrix, possessing numerous binding sites for various extracellular matrix proteins and integrins.

A significant quantity of cytoplasmic proteins is either directly associated with integrins (e.g., talin) or are locally recruited in proximity to integrins (e.g., vinculin, p130cas, zyxin, and filamin A) [27]. These proteins collectively form a focal adhesion complex that is connected to the actomyosin cytoskeleton. Talin and p130cas experience tension-induced unfolding, leading to the exposure of supplementary binding sites, including vinculin-binding sites in talin [28], or alterations of binding sites due to Src-mediated phosphorylation of p130cas [29]. Research employing a Forster resonance energy transfer (FRET) tension sensor (TsMod) has shown that vinculin experiences tension at focal adhesions [30]. Consequently, ECM–integrin adhesion is augmented through the recruitment and alteration of proteins that aggregate integrins and enlist the actomyosin cytoskeleton [31]. Only a fraction of these complexes may be stabilized in response to applied force. Other actin-associated protein–protein interactions may be classified as “slip bonds”, which dissociate under applied force, resulting in the breakdown of protein complexes and localized dissipation of stresses.

The force-induced reconfiguration of focal adhesion complexes leads to alterations in the control of Rho family small GTPases. For instance, the Rac-GAP FilGAP is released from filamin A in a tension-dependent way and may locally diminish Rac-regulated actin and membrane activity [32]. In contrast, the Rho-GEFs LARG and GEF-H1 are stimulated by force exerted on integrins and may locally enhance Rho-dependent actomyosin contractility [33]. Modifications in the organization and contractility of the actin cytoskeleton facilitate both short- and long-range force transmission to downstream destinations.

Short-range transmission modifies the arrangement of focal adhesions and the adhesion strength of cells to the extracellular matrix [34], while long-range transmission influences cell morphology, destiny, and functionality. A primary objective is the nucleus and the modulation of gene expression [35,36]. All components of the cytoskeleton are associated with a protein complex known as the linker of nucleoskeleton and cytoskeleton (LINC) complex, which connects the nuclear envelope [37].

This complex facilitates force transmission across the nuclear envelope, leading to nuclear deformation and modifications in chromatin structure and organization that influence gene expression [38,39]. Several muscle tissue illnesses, including muscular dystrophy and cardiomyopathies, arise from mutations in components of the LINC complex. The interaction between the nucleus and cytoskeleton is crucial for cell migration, wound healing, cancer metastasis, and development [40,41].

### 2.2. Role of Integrins in Mechano-Signal Transduction

Integrins play a primary role in mechano-transduction. Being cell surface receptors, they sense mechanical cues from the extracellular environment as extracellular mechanical signals. Integrins transfer these extracellular signals within the cell, where they become a biochemical signal. These mechanical cues might be hydrostatic pressure, fluid shear stress, tensile stress, and stiffness and viscosity of ECM [2]. In this way, integrins serve as mechanoreceptors and sensors of mechanical changes in ECM. The mechanical stress in ECM induces conformational changes in integrins leading to their activation. These conformational changes enhance the binding affinity of integrins towards ECM ligands, thereby strengthening their interaction with ECM, which in turn activates intracellular signaling. This process is called outside–in signaling mediated by integrin [1,42].

Mechanical stimulation by hydrostatic pressure is a key factor in regulating cellular responses through mechano-signal transduction, with integrins and talin playing critical roles. Hydrostatic pressure, a force exerted by fluids within biological environments, induces mechanical stress on cells. Integrins act as transmembrane receptors that link the extracellular matrix (ECM) to the cytoskeleton, translating external physical forces into intracellular biochemical signals [43]. When cells are subjected to hydrostatic pressure, integrins bind to ECM ligand proteins, triggering integrin clustering on the cell membrane. This clustering recruits talin and focal adhesion proteins, such as kindlin and vinculin, to form focal adhesions (FAs). Talin is mechano-sensitive and directly connects integrins to the actin cytoskeleton, transmitting mechanical stress to the cytoskeleton [44].

This mechanical signal triggers downstream signaling cascades, including the phosphorylation of mitogen-activated protein kinase (MAPK) and phosphoinositide 3-kinase (PI3K), which regulate cell behaviors like migration, proliferation, and survival. Hydrostatic pressure’s influence extends beyond signal initiation to cellular mechanics and structure. Talin strengthens integrin–cytoskeleton connections, enhancing cellular resistance to deformation under pressure [45]. It also mediates cytoskeletal tension and facilitates the mechanical balance to preserve cell shape. Moreover, positive hydrostatic pressure triggers potassium channel activity, while negative pressure activates cytoskeletal components like the sodium–hydrogen exchanger 1 (NHE1), creating dynamic intracellular adaptations [46]. This process ensures cells respond effectively to pressure changes during physiological functions such as embryonic development and tissue repair [44]. Integrins and talin orchestrate a finely tuned mechano-transduction system, translating hydrostatic pressure into precise cellular responses that maintain tissue integrity and adapt to environmental demands.

Furthermore, integrins are involved in the formation of focal adhesion sites by recruiting different proteins, which are responsible for triggering downstream intracellular signaling pathways. These pathways mainly include PI3K/AKT, MAPK/ERK, and RhoA pathways, which promote cell proliferation, survival, and migration. Cell proliferation, survival, and migration are vital for tissue repair in reaction to mechanical stress [2,47]. In this way, integrins play a vital role by sensing mechanical stress and producing the ultimate response to it by triggering mechano-signal transduction involving a complex network of different signaling pathways.

### 2.3. Integrin-Associated Proteins

Integrin-associated proteins play an integral role in mechano-signal transduction. They link integrins with cytoskeletal elements and a complex network of different signaling pathways happening in the cytoplasm. In this way, they transfer the mechanical signal from integrin to the cytoplasm, thereby integrating the mechanical signal into a complex of biochemical intracellular signaling pathways. The cytoplasmic domains of integrin are directly associated with various cytoplasmic and cytoskeletal proteins [48]. The fundamental integrin-associated proteins involved in mechano-signal transduction include focal adhesion kinase (FAK), c-Src, talin, vinculin, paxillin, kindlins, integrin-linked kinases (ILK), alpha-actinin, filamin, tensin, and zyxin [5,49,50,51,52,53,54].

Integrin- and proteoglycan-mediated adhesions connect to the actin cytoskeleton at focal adhesions. Many different types of enzymes, including kinases, phosphatases, proteases, and lipases, as well as scaffolding molecules and GTPases, make up focal adhesions [55]. The location, size, and composition of focal adhesions within cells characterize their classification. Prior to the formation of larger focal adhesions, regulated by Rho activity, at the periphery of spreading or migrating cells, Rac and Cdc42 regulate small focal adhesions, also known as focal complexes [56,57]. Both peripheral and central focal adhesions, linked to stress fiber ends, are observed in cells cultivated on two-dimensional (2D) stiff surfaces. Although there have been recent attempts to classify focal complexes and adhesions into subsets [58], the exact features that differentiate a focal complex from an adhesion remain unclear. In particular, fibrillar adhesions comprise α5β1 integrin and tensin, and they develop by the extension of focal adhesions [59]. Previous studies have focused on cells sticking to inflexible two-dimensional surfaces; however, 3D matrix adhesions have been established for fibroblasts clinging to collagen gels and matrices produced by 3D cells, as well as for epithelial cells in 3D collagen [55,60]. With the help of cytoskeletal proteins that aggregate into massive supramolecular complexes, cells adhere to one another and to the extracellular matrix [61].

The adaptor protein vinculin plays a crucial role in regulating FAs [62]. As opposed to wild-type cells, cells lacking vinculin exhibit decreased adhesion to many ECM proteins, faster migratory rates, and fewer and smaller adhesions [63]. The chemical mechanisms via which vinculin exerts its different effects on cell adhesion and motility remain poorly understood, despite vinculin’s profound importance in these processes. A vinculin structure consists of three primary domains: a head domain at the N-terminus, a neck area that is flexible and rich in proline, and a tail domain at the C-terminus [64]. These domains undergo conformational rearrangements, which activate vinculin. Vinculin is restricted to an inactive conformation in the cytoplasm as a result of intramolecular interactions between its head and tail domains [65,66].

When vinculin is recruited to FAs, its structure changes to an active, open conformation. Actin, phosphatidylinositol (4,5)-bisphosphate (PIP2), and paxillin are required for full access and direct interaction with the head, neck, and vasodilator-stimulated phosphoprotein, talin, and α-actinin, respectively, during this activation process [62]. It is unclear, however, the potential impact of these connections on FA production, as most research on vinculin interaction sites has utilized biochemical experiments that use isolated proteins.

Researchers have discovered that vinculin must interact with talin before it can trigger the creation of FAs. Integrins are directly impacted by the interaction between the opened and activated form of vinculin and talin, which causes them to cluster in an active conformation and causes FA expansion. After vinculin appears in FAs, paxillin is recruited, but this happens regardless of the paxillin-binding location in the vinT domain of vinculin. A key connection between FAs and the actin network appears to be vinT, according to the data [67]. Focal adhesions are stabilized, and integrin clustering and expansion are enhanced when vinculin is recruited to talin. Hence, vinculin controls integrin activation directly via talin, according to recent research [68].

The impact of talin on integrin functionality is extensive. It transmits signals through integrins in both inside–out and outside–in orientations, and it also affects the arrangement of the actin network and the makeup of focal adhesions [69]. Recent research on talin has highlighted its distinct function in the inside–out signaling of integrins, namely their transition from a basal or “resting” state to a more “active” one, enabling more effective engagement with their corresponding ECM ligands (integrin activation). There is limited knowledge regarding the function of talin in the interaction between integrins of the same or distinct subfamilies, as well as with other signaling pathways [70].

FAK and c-Src are members of the family of non-receptor tyrosine kinases. They play a key role in mechano-signal transduction by activating other molecules involved in mechano-signal transduction and linking them with integrins. FAK plays a very important role in mechano-signal transduction by linking integrins to cytoskeletal elements. Cross-linking cytoskeletal elements with integrin, talin, and kindlin is pivotal in mechano-signal transduction [71,72]. Vinculin acts as a cross-linker between talin and actin filaments and contributes to mechano-signal transduction [73]. Similarly, paxillin is involved in mechano-signal transduction by linking F-actin cross-linkers, i.e., talin and kindling, with the cytoplasmic tail of β integrin, in response to an external mechano-signal [72]. ILK links and modulates the cytoskeleton with integrin by interacting with PINCH and Parvin proteins in response to mechanical signals [74]. By mechanosensing upon integrin activation, α-actinin binds with integrin, actin filament, and vinculin. This interaction activates vinculin to reinforce the interaction with actin filaments of the cytoskeleton with integrins via α-actinin [75,76]. Filamin is also an integrin-associated protein that cross-links the cytoskeleton with the cytoplasmic tails of integrins [77]. Tensin and Zyxin are also mechanosensitive proteins, which, upon integrin-sensed mechanical cues, regulate the cytoskeletal elements and gene expression [78,79,80]. In this way, different integrin-associated proteins are involved in transferring the mechanical signals perceived by integrin to the cytoskeleton and contribute to mechano-signal transduction to produce an ultimate response.

## 3. Mechano-Signal Transduction Mediated by Integrins

Being cell surface receptors, integrins are the fundamental players in mechano-signal transduction. It senses the physical cues from ECM and transfers them within the cell in the biochemical signal form where it reaches the target proteins, and the ultimate cell response is generated [81]. Mechanical changes in the ECM cause integrins to bind with the ligand. Consequently, the conformation of integrins changes into active form. This integrin activation induces the clustering of integrin molecules on the cell membrane. The integrins in their active form recruit cytoplasmic proteins, including FAK, c-Src, talin, vinculin, and paxillin, etc., at the cytoplasmic tail of the β-subunit of integrin and generate a large molecular complex called focal adhesion (FA) (Figure 1 asterisks: **).

An important biological function of integrin–ligand interactions is the regulation of signal transduction by the binding event. Because cells constantly assess their pericellular surroundings and react by swiftly shifting their location and differentiation status, adhesion is extremely dynamic and necessitates a receptor activation mechanism that is highly responsive. Since integrins do not have any enzyme activity, the cytoplasmic surface of the plasma membrane is where signaling complexes are assembled, which in turn induces signaling [82]. Despite claims to the contrary, multiple lines of evidence point to the fact that primed integrins are stretched and bent integrins are inactive (Figure 1 asterisk: *). Integrin was bent at an angle of 135° in the first crystal structure of αVβ3. Receptors produced on cell surfaces are unable to bind ligands when integrins are locked in this condition using disulfide bond engineering [83]. Bent structures were the most common when electron microscopy was used to examine the gross structure of integrins in environments with low ligand binding, such as in buffers containing Ca^2+^ or after intersubunit covalent bond constraints were introduced [84]. Although ligand engagement may be hindered in bent integrins due to the orientation of the ligand-binding pocket toward the plasma membrane, a “breathing” movement at the juxtamembrane domain may allow the conversion of bent to extended integrin [83,85]. Regarding this matter, according to Adair and Yeager [86], a cryo-electron microscopy analysis of unstimulated αIIbβ3 revealed a partly stretched shape. Stimulatory mAb binding may subsequently cause activation by shifting a conformational equilibrium.

When the connections between the α and β cytoplasmic tails are severed, the leg regions stop to communicate, the head and legs separate from one another, and the head recedes from the surface of the cell. This concept is strongly supported by the large number of stimulatory mAb epitopes that have been found to reside in the knee or leg areas [87] and by investigating soluble recombinant integrins using electron microscopy [83]. When the integrin is bent, these epitopes are covered, but when it is extended, they become significantly exposed [85]. Although our knowledge of the conformational changes that take place during unbending is relatively limited, it appears that the mobility of the hybrid domain is a crucial component. From its intracellular position, the integrin is transported to its ligand-binding site by this domain. Electronic microscopy revealed a sharp angle between the hybrid domain and β-I in the bent state, but an obtuse angle in the stretched, ligand-occupied state [83]. It is probable that unbending is an essential prerequisite for the mobility of the hybrid domain, as the β-I domain is immobile in relation to the hybrid domain when it is bent. Affinity control relies on hybrid domain migration, according to several lines of evidence. The α subunit β-propeller is located close to the hybrid domain’s activating mAb epitopes [88]. These previously covered epitopes by bending integrins become apparent when the hybrid domain is swung away from the propeller. In addition, integrins can be activated by the design of glycosylation sites between the hybrid domain and the β-I-domain [89]. A curved conformation may be possible for ligand-bound integrin, contrary to what is currently believed about integrin function, which implies that extension is necessary for conversion to a high-affinity receptor. The bent conformation of crystallized αVβ3 has the potential to bind a cyclic RGD peptide [90], and electron microscopy images of complexes with fibronectin fragments also reveal bent αVβ3 [86].

FAs play an important role in mechano-signal transduction because they serve as a platform for mechano-transduction within the cell with different adaptor proteins for it. The proteins at the focal adhesion site interact and associate the cytoskeletal elements with integrin, which in turn connects the cytoskeleton to the ECM or extracellular environment. At FAs, integrins directly sense mechanical signals from ECM and transfer them in the form of biochemical signals to the cytoskeleton through integrin-associated proteins. Interestingly, the integrin-associated cytoplasmic proteins also serve as adaptor proteins, which, in a scaffolding manner, recruit further signaling proteins to the integrin and initiate a downstream signaling cascade. These signals reorganize the cytoskeleton, which in turn stimulates a complex network of signaling pathways along with gene expression. These signaling pathways and gene expressions impact cell behavior in accordance with external physical cues [91].

FAs are formed in response to mechanical stress in ECM. It monitors the strength of that extracellular mechanical stress. Focal adhesion sites linking integrin to the cytoskeleton mediate the transfer of mechanical stress to the cytoskeleton, which in turn reorganizes to a tense state [92,93,94]. These cytoskeletal tensions are transferred to ECM via integrin in the form of force. Consequently, integrins monitor ECM stiffness by sensing the resistance of ECM to this force. If the resistance of ECM is high, then stiffness is high, which would be sensed by integrins, and FA is reinforced via outside–in signaling, and the cytoskeleton becomes tenser. The integrin-sensed ECM’s high stiffness may also result in the expression and secretion of proteins that model the ECM as less stiff to meet the biomechanical demands. On the other hand, if ECM stiffness is low, then the integrin clustering becomes lowered, and they become less active, resulting in FA becoming weak and the cytoskeletal becoming less tense. This adaptation is significant in permitting cell integrity, proliferation, differentiation, and wound healing. Any disruption in this adaptation may result in different disorders such as cancer, tissue deformities, fibrosis, etc. Thus, integrin mediates the cell and ECM adaptation to meet the demands [47,95,96].

Mechano-signal-mediated integrin activation stimulates FAK via auto-phosphorylation [97]. Activated FAK in turn phosphorylates and interacts with c-Src and forms the FAK-Src complex. This complex serves as a significant pivot for different signaling pathways resulting in cell proliferation, survival, and migration [98]. These pathways include MAPK/ERK, Jnk, RhoA/ROCK, PI3K/AKT, and YAP/TAZ pathways [99].

**Figure 2:**
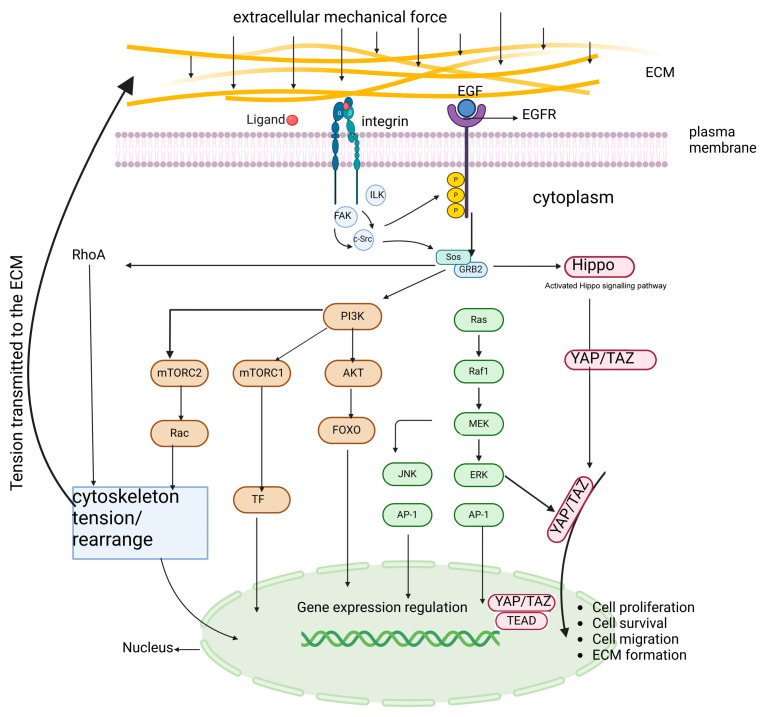
Pathway of integrins and its associated protein-mediated mechano-signal transduction. Integrin-mediated mechano-signal transduction involves FAK activation leading to RhoA signaling. RhoA signaling promotes actin assembly, mediating cell contractility with tensed cytoskeleton. Cytoskeleton tension inhibits the hippo pathway-mediated phosphorylation of YAP/TAZ. Resultantly, the dephosphorylated YAP/TAZ enters the nucleus and associates with TEAD transcription factor and regulates the expression of genes involved in cell proliferation, survival, migration, and ECM production. Integrin mediates the mechano-signal transduction by influencing gene expression via YAP/TAZ to produce cell response to extracellular biomechanical signals. TF: Transcription factor. Created in https://BioRender.com.

Integrin-mediated mechano-signal transduction involves FAK activation leading to RhoA/ROCK signaling. RhoA/ROCK signaling promotes actin assembly, mediating cell contractility with a tensed cytoskeleton. Cytoskeleton tension inhibits the Hippo pathway-mediated phosphorylation of YAP/TAZ. Resultantly, the dephosphorylated YAP/TAZ enters the nucleus and associates with the TEA domain (TEAD) transcription factor and regulates the expression of genes involved in cell proliferation, survival, migration, and ECM production [91,100]. When integrins are ligated (bound to their ligands), LATS is inhibited. This inhibition allows YAP to translocate to the nucleus. This suggests that LATS normally acts to prevent YAP from entering the nucleus, but when integrins are activated, LATS is inhibited, allowing YAP to enter the nucleus and potentially activate gene expression [101,102,103]. This signaling pathway is dependent on both Src and Rac. This suggests that these two proteins are required for the inhibition of LATS and subsequent nuclear translocation of YAP. The above information concerning the pathway of integrins and their associated protein-mediated mechano-signal transductions is summarized in Figure 2.

## 4. Integrin-Associated Protein-Mediated Mechano-Signal Transduction

### 4.1. Focal Adhesion Kinase (FAK)

FAK is an integrin-associated mechano-sensitive protein. It is a non-receptor tyrosine kinase. In the cytoplasm, it exists in an auto-inhibited form. It consists of three PPR domains alternating with three major domains. Three major domains are named FERM, kinase, and FAT. The FERM domain is located at the N-terminus, while the FAT domain is at the C-terminus. AT PPR 1, the tyrosine 397 residue is present, which is very crucial for the activation of FAK [104].

Upon mechano-signal reception from ECM, integrin becomes activated. The activated integrin further activates FAK via auto-phosphorylation of tyr 397. After autophosphorylation, FAK recruits Src, which detaches the FERM domain from the kinase domain, and FAK becomes fully active. It undergoes auto-phosphorylation and starts to play its role in signal transduction by interacting with and activating other integrin-associated proteins, including talin and paxillin [105,106]. Consequently, these activated proteins trigger a complex network of signaling pathways, including MAPK/ERK, PI3K, and RhoA/ROCK, resulting in cell proliferation, survival, and migration, respectively.

### 4.2. c-Src

c-Src is also one of the mechano-sensitive integrin-associated proteins. It is a non-receptor tyrosine kinase. It exists in auto-inhibited folded form in the cell. It mainly consists of four Src homology (SH) domains, named SH1, SH2, SH3, and SH4 domains. SH1 domain has kinase activity. Between the SH2 and SH1 domains, the SH2-Kinase linker is present, which interacts with the SH3 domain in an auto-inhibited state of c-Src. The c-Src molecule has a tyr 527 residue towards the C-terminus and a tyr 416 residue at the SH1 kinase domain. Both tyr 527 and tyr 416 play an important role in c-Src activation. In the auto-inhibited state, the SH2-kinase linker interacts with the SH2 domain, while phosphorylated tyr 527 interacts with the SH2 domain. This interaction results in a folded and thus auto-inhibited conformation with the kinase domain having a dephosphorylated tyr 416 residue [107]. When FAK is phosphorylated, it provides binding sites for the SH2 domain of Src [108,109]. This interaction is critical because it effectively activates Src. The recruitment of Src to FAK involves the binding of the SH2 domain of Src to phosphorylated tyrosine residues on FAK. This recruitment can induce a conformational change in Src, prompting the release of the SH2 domain from the C-terminus of Src, allowing it to adopt a more open, active conformation [110].

Mechano-signal-mediated active integrins activate FAK. The active FAK interacts with c-Src at its SH2 domain. This interaction between FAK and c-Src recruits’ phosphatases, which dephosphorylate tyr 527 of c-Src, leading to disruption of the interaction between tyr 527 and the SH2 domain of c-Src. The recruitment of FAK also induces auto-phosphorylation of tyr 416 at the kinase domain. The auto-phosphorylation at tyr 416 induces conformational changes to the kinase domain, leading to its activation. Once c-Src is activated in response to integrin activation, it starts to phosphorylate other integrin-associated proteins, including paxillin and vinculin [111]. c-Src also contributes to the activation of the downstream signaling pathways, including the MAPK/ERK, PI3/AKT, and RhoA/ROCK pathways. Thus, c-Src serves as an integrin-associated mechano-sensitive protein, which plays an important role in mechano-signal transduction by modulating different signaling pathways to produce cell response.

### 4.3. Talin

Talin is a mechano-sensitive integrin-associated protein. It mediates the link between actin and integrin at the FA site upon extracellular mechanical signal by integrins. It consists of the N-terminus FERM domain, which interacts with the cytoplasmic tail of β-integrin. Talin has also thirteen α-helices toward the C-terminus, organized in the forms of beads, which interact with F-actin. The biomechanical integrin activation induces the FAK-mediated activation of talin. Active talin from its FERM domain binds with the cytoplasmic β-tail of integrin, while α-helices interact with F-actin [53]. Thus, talin serves as the simplest cross-linker for integrin and cytoskeleton. The intraspinal nature of talin refers to its structural and functional roles within cells. Talin has a multidomain structure, composed of a large head domain and a long rod-like tail. This allows talin to interact with various proteins, including integrins, actin filaments, and other signaling molecules, making the linkage between the ECM and the actin cytoskeleton. Talin is critical for the activation of integrins, which are transmembrane receptors that mediate cell–ECM adhesion. Integrin activation and increased cell adhesion result from talin binding to its cytoplasmic domain, causing conformational changes [112]. The ability of talin to stretch enhances its binding sites for actin and vinculin, thus reinforcing the actin–integrin complex connection.

### 4.4. Vinculin

Vinculin is another mechano-sensitive protein. In the cytoplasm, it remains in auto-inhibited form. However, integrin activation via extracellular biomechanical signal induces FAK activation, which in turn phosphorylates and activates paxillin. Phosphorylated paxillin interacts with auto-inhibited vinculin. Paxillin interaction converts the closed conformation of vinculin into an open active conformation. The activated vinculin cross-links the actin filaments to the talin. In this way, the talin–vinculin axis connects the cytoskeleton to integrin in response to extracellular biomechanical signals [113]. It has also been reported that vinculin becomes activated by undergoing c-Src-mediated phosphorylation at tyr 100 and tyr 1065. After activation, it interacts with talin and transmits the cytoskeletal tensions to the ECM via integrin and plays a role in inside–out signaling in mechano-transduction [114]. FAK is essential for paxillin-dependent vinculin activation [115]. Paxillin serves as a docking protein at focal adhesions, where it interacts with various proteins, including FAK [116]. When FAK is activated, it phosphorylates paxillin, which then facilitates the recruitment and activation of vinculin [117]. This process is essential for the control of cell adhesion and signaling in response to extracellular signaling. The vinculin activation enhances the stability of focal adhesions and plays a vital role in cell migration and mechanical signaling.

### 4.5. Paxillin

Paxillin serves as an integrin-associated mechano-sensitive protein. It exists in the cytoplasm in auto-inhibited form. Upon biomechanical integrin activation, FAK becomes activated. FAK phosphorylates and activates paxillin [118]. Paxillin is pivotal to the mechano-signal transduction as it, via its N-terminus LIM domains, mediates the interaction of kindling and talin, attached with F-actin, to the cytoplasmic tails of β-integrin. It also mediates F-actin polymerization by recruiting different molecules responsible for downstream signaling cascades at its N-terminus. This signaling cascade activates actin polymerization [72]. Thus, paxillin functions as a mechano-sensitive protein by recruiting other molecules to integrin tails and modulating F-actin polymerization and cytoskeleton tension.

### 4.6. Kindlins

In the cytoplasm, kindlin remains in inactive form. As a result of extracellular biomechanical integrin stimulation, FAK is activated, which in turn activates kindling. The activated kindling interacts with itself and mediates the interaction of other linker mechanosensitive proteins to β-integrin tails [72,119].

### 4.7. Integrin-Linked Kinases (ILK)

ILK is a mechano-sensitive integrin-associated protein. Upon extracellular biomechanical signal activation, c-Src is activated, which in turn activates ILK. Activated ILK then interacts with the cytoplasmic tail of β-integrin. It interacts with actin filaments carrying α-actin and filamin to the cytoplasmic tail of β-integrin via β-parvin and Mig2, respectively. In this way, it connects the cytoskeleton with the biomechanically activated integrins and contributes to mechano-signal transduction. It is also pivotal to the Akt pathway because it activates phosphorylates Akt, which then triggers the downstream Akt pathway promoting cell proliferation, growth, and survival [120].

### 4.8. Filamin

It is also a mechano-sensitive protein. Upon extracellular biomechanical tension-mediated integrin activation, filamin obtains active conformation. The activated filamin acts as a cross-linker of F-actin filaments to the cytoplasmic tails of mechanically activated β-integrin. It also serves as a hub for many downstream signaling pathways, where it serves as a scaffolding protein [49].

### 4.9. ICAP-1

Various physiological processes, such as cell survival, differentiation, migration, proliferation, and metabolism, are regulated by the IACs through numerous signal transduction pathways [121]. A novel binding protein for the cytoplasmic region of β1 integrin, ICAP-1α, has been early characterized in a study. The involvement of ICAP-1α in integrin-dependent cell adhesion is supported by two observations: firstly, its binding to a conserved region of the β1 integrin cytoplasmic domain, which is essential for integrin adhesive function and focal contact localization, and secondly, the regulation of ICAP-1α phosphorylation during interactions between cells and their respective matrices.

Cell adhesion and cytoskeletal rearrangement are likely facilitated by structural and regulatory proteins with overlapping binding sites in the cytoplasmic domains of integrins. The discovery of ICAP-1α and the definition of the β1 integrin cytoplasmic domain sequences required for ICAP-1α binding are anticipated to improve future studies on the control of particular integrin-dependent cellular events [122].

### 4.10. SHARPIN

Through its direct interactions with diverse proteins, SHARPIN modifies target molecules through linear ubiquitination and contributes to cell proliferation, immunology, inflammation, and collagen regulation [123,124]. Because it is a multifunctional junction protein, the exact biological role of SHARPIN is still unknown. There is a decrease in collagen synthesis and secretion, as well as in the assembly of extracellular collagen fibers and the contraction of collagen gels due to insufficient cellular traction, in the mammary stromal fibroblasts of SHARPIN-deficient mice [125]. The inflammation in the liver was worsened when the expression of SHARPIN was silenced in the livers of mice, resulting in an increase in TNF-α, MCP-1, IL-1β, and IL-6 in the liver tissue. However, the levels of collagen mRNA and profibrogenic cytokines were not different between wild-type and knockdown animals, indicating that the process of liver fibrosis is unclear [126].

### 4.11. Negative Regulator of Integrin

Integrins are critically regulated by several proteins that modulate their activity to ensure proper cellular function, among which filamin [122,127], ICAP (Integrin Cytoplasmic Domain-Associated Protein-1) [122,128], and SHARPIN [129] play essential roles as negative regulators. Filamin links integrins to actin filaments. It is involved in mechano-transduction processes [122]. However, its negative regulatory role is revealed by its ability to bind to integrin cytoplasmic tails, thus inhibiting integrin activation by preventing association with integrin activators such as talin [127]. ICAP-1 specifically interacts with the β1 integrin tail, competing with talin binding, which is critical for integrin inside–out activation, thereby maintaining integrins in an inactive state. In addition, SHARPIN is part of the linear ubiquitin chain assembly complex (LUBAC) [129,130] and has been recognized for its ability to inhibit integrin activation by blocking talin binding to integrins and negatively regulating integrin-linked kinase (ILK) pathways [129]. Together, these proteins contribute to the fine-tuned control of integrin activation and signaling. They balance cellular adhesion and migration processes critical for tissue integrity and immune responses [131].

## 5. Downstream Signaling Pathways Mediated by Integrin-ECM Axis

The integrin-ECM axis is fundamental in integrin-mediated mechano-signal transduction. It serves as a hub for multiple downstream signaling pathways during mechano-signal transduction. When a biomechanical signal is perceived by integrin, they bind with ligands like fibronectin, collagen, vitronectin, osteopontin, and laminin, etc. This ligand binding to the integrin extracellular domain induces their activation. The activated integrin leads to the activation of multiple molecules, which scaffolds and contributes to multiple downstream pathways.

The biomechanically activated integrins activate FAK, which in turn activates c-Src. Both FAK and c-Src reinforce their activity and serve as a hub for multiple downstream signaling pathways. Thus, the ECM-integrin axis during mechano-signal transduction activates the FAK/Src signaling pathway [132]. During mechano-transduction, the integrin-ECM axis also activates ILK. Activated ILK in turn activates such molecules, which trigger the downstream signaling pathways involved in focal adhesion, thereby contributing to focal adhesion signaling. In the presence of extracellular mechanical stress, the FA signaling results in cytoskeletal tensions. The cytoskeleton tensions cause the activated FAK to trigger a complex network of multiple downstream signaling pathways, which promotes cell survival, proliferation, differentiation, and migration [2].

The integrin-ECM axis also plays a pivotal role in the activation of other receptor tyrosine kinases, e.g., epidermal growth factor receptor (EGFR), which then bind with the epidermal growth factor through its extracellular domain. This causes the recruitment of Grb2 protein to SOS, which in turn activates Ras via its GTP loading. The active Ras stimulates RAF, which then recruits MEK, which ultimately leads to ERK activation through its phosphorylation. ERK enters the nucleus and stimulates transcription factor AP-1, which allows the transcription of genes promoting cell proliferation and survival [133]. Furthermore, activated receptor tyrosine kinase activates the PI3K/Akt pathway by facilitating Grb2 recruitment to SOS. Akt activates FOXO, which promotes the transcription of pro-survival genes. Activated MEK also activates JNK, which then stimulates c-Jun. In this way, integrin and receptor tyrosine kinase cooperate with each other and trigger downstream signaling pathways [134].

## 6. Downstream Pathways Mediated by FAK-Src Axis

Integrin activation causes FAK activation via auto-phosphorylation, creating a docking site for c-Src on it. It phosphorylates and recruits c-Src and forms the FAK/c-Src complex. This axis plays a key role in integrin-mediated signal transduction by activating other molecules involved in multiple downstream signaling pathways [132].

FAK-Src complex triggers ERK, JNK, Akt, and mTOR pathways by activating Grb2, which in turn activates and recruits SOS protein. This Grb2 and SOS complex activates Ras, thus further activating RAF. Resultantly, RAF activates MEK, which in turn activates both JNK and ERK. JNK activates c-Jun, which joins with other transcription factors like AP1 and promotes the transcription of pro-survival genes [134,135]. While on the other hand, ERK activates the AP-1 transcription factor and promotes the transcription of pro-survival genes [134]. FAK, after recruiting c-Src, interacts with and activates PI3K, which in turn phosphorylates and activates Akt, resulting in activation of FOXO. Consequently, FOXO transcribes the pro-survival genes. Furthermore, Akt also activates the mTORC [132,134]. It is reported that Akt activates both mTORC1 and mTORC2 and contributes to the triggering of the mTOR pathway. In turn, mTORC2 activates Rac, which rearranges the cytoskeleton. On the other hand, mTORC1 activates p7056k, which further activates different transcription factors involved in the expression of pro cell survival genes [98].

FAK-Src plays a pivotal role in the Rho/GTPase signaling pathway, activating Rho GTPase. Active Rho GTPase produces cytoskeleton tensions by rearranging the actin cytoskeleton. This cytoskeleton tension inhibits the Hippo pathway and activates YAP and TAZ via their dephosphorylation. Consequently, active YAP/TAZ translocate to the nucleus, where they associate with the TEAD transcription factor and facilitate the transcription of genes that favor cell proliferation, survival, and migration [136,137]. In this way, in integrin-mediated mechano-signal transduction, the FAK-Src axis plays a hub for different downstream signaling pathways, resulting in the ultimate cell response to the extracellular cues.

## 7. Importance of Integrin and Its Associated Proteins as a Mediator for Mechano-Signal Transduction

Integrin and many of its associated proteins play a fundamental role in mechano-signal transduction. Any malfunction in integrin or in its associated proteins may lead to disruption of mechano-signal transduction [3]. Mechano-signal transduction is a process that involves the reception and conversion of mechanical signals from the cell’s external environment into intracellular biochemical signals and produces a response to the mechanical signal. In this context, mechano-signal transduction is very important for the cell’s physiological functions in accordance with extracellular biomechanical cues. These physiological functions may include wound healing, cell differentiation into a tissue, developmental processes, and immune responses involving cell proliferation, differentiation, and migration [7,138].

Integrins are transmembrane proteins with extracellular, transmembrane, and cytoplasmic parts. They directly sense the biomechanical signal from the extracellular matrix and bind with ECM ligands via their extracellular binding sites. Moreover, it recruits and activates different associated proteins, including FAK and c-Src, which are pivotal to FA formation. Consequently, they serve as the most basic mechano-sensitive protein in mechano-transduction. It has been reported that disruption of FAK disrupts FA and causes apoptosis by the activation of caspase-3 [6]. Similarly, it has been investigated that inhibition of Src leads to failure of MAPK signaling upon mechanical stress in myogenic precursor cells [139].

Briefly, being responsible for downstream signaling pathways and producing a cell response to mechanical stress, integrin-associated proteins are vital for mechano-signal transduction. In this regard, integrins are also pivotal because most of the mechano-sensitive proteins involved in mechano-signal transduction are associated with integrins. They require biomechanical signal reception and activation of integrin for their own activation.

## 8. Pathologies Resulting from Malfunctioning of Mechano-Signal Transduction

Integrin-mediated mechano-signal transduction is vital for the maintenance of extracellular mechanical cue-mediated cell homeostasis. Integrin-mediated mechano-signal transduction results in cell response, which usually favors cell proliferation, survival, migration, and differentiation. Therefore, it is linked with tissue differentiation, developmental process, and wound healing. Commotion in mechano-signal transduction may result in pathologies linked with altered cell proliferation, survival, and differentiation. Different types of cancer may arise from integrin-mediated signaling. In cancer, dysregulation of integrin signaling causes excessive cell proliferation, migration, and survival, which in turn favor tumorigenesis and metastasis. The YAP/TAZ pathway is activated in integrin-mediated mechano-transduction. It has been reported that in cancers, the YAP/TAZ pathway favors the expression of genes that promote metastasis, tumorigenesis, and resistance to immunotherapy [124]. Excessive and prolonged extracellular mechanical stimulation leads to deregulated integrin and integrin-associated protein-mediated mechano-signal transduction. Furthermore, the abnormal expression of integrin and integrin-associated proteins also results in deregulated mechano-signal transduction [140]. There are various other pathologies that result from deregulated integrin-mediated mechano-signal transduction, including fibrosis of different organs, auto-immune and inflammatory disorders, cardiovascular disease, and carcinoma. Therefore, the proper regulation of integrin-mediated mechano-signal transduction is essential for proper cell function.

Loss of normal mechano-signal transduction of cells manifests in pathological states such as cancer. The existence of (distant) metastases presents the main cause of mortality caused by cancer. According to the references, low tumor cell adhesiveness or detachment is primarily the result of loss of inhibition of cell–cell contact and the loss of function of key proteins involved in mechano-signal transduction, followed by tumor cell migration and invasion during metastasis.

The leading cause of cancer-related death and disability is metastasis, which is the spread of cancer cells from the primary tumor to nearby tissues and even other organs [141]. Approximately 90% of cancer deaths are expected to be caused by metastasis [142]. The steps of metastasis are sequential and interdependent. The last step in the metastatic cascade is for cancer cells to invade and proliferate in other organs, which involves them separating from the primary tumor, entering the bloodstream and lymphatic systems, evading the immune system, and then extravasating at peripheral capillary beds [143].

Visible, cancerous secondary tumors develop when metastasis-causing cells foster angiogenesis and proliferation in the surrounding microenvironment. The majority of cancer deaths are due to systemic metastasis, yet most cancer studies do not examine metastasis in living organisms [144]. Metastatic cells, according to the epithelial–mesenchymal transition (EMT) theory, develop from epithelial stem cells or differentiated epithelial cells. This happens because of a series of genetic changes that transform the cell into a tumor cell with mesenchymal traits [145]. The idea behind this approach is based on studies showing that many types of cancers start in epithelial tissues, which are defined by the breakdown of cell–cell and cell–matrix interactions as the tumor grows. Neoplastic cells eventually develop into something that looks like mesenchymal cells but has less cell–cell adhesion, a dysmorphic shape, and can spread to other organs [146].

Among the many mechano-transduction diseases, cancer is perhaps the most intriguing. Rapid changes in the mechanics of the extracellular matrix (ECM), remodeling of the ECM, and the resulting disturbance of cytoskeletal tension and mechano-transduction signaling have been recognized as important factors in cancer, metastasis, and malignant transformation in the past decade [147]. In addition to genetic abnormalities and increased oncogene activity, cytoskeletal reorganizations—particularly alterations in the tensional force generated by the actin–myosin apparatus—play a critical role in the morphological changes that tumor cells undergo when they develop invasive characteristics. One of the main regulators of cytoskeletal tension is the Rho family of GTPases.

To regulate the phosphorylation of myosin-II light chains, Rho acts via Rho kinase (ROCK II), which phosphorylates myosin phosphatase and inhibits it. Cytoskeletal tension significantly affects signaling pathways linked to cancer progression, even though there are conflicting results concerning Rho activity in tumors. Some studies have shown increased Rho activity and cytoskeletal tension, while others have found decreased Rho activity in solid tumors [148]. Furthermore, extracellular matrix stiffness affects tumor cytoskeletal tension, according to multiple studies [148]. The physical environment of cancer cells within the tumor and the normal cells around it is affected by changes in tissue stiffness, tumor growth as a result of cell proliferation, and/or elevated interstitial fluid pressure [149].

Through mechano-transduction, this altered physical milieu can affect the fate of these cells. Mammary epithelial cells can invade cystic lumens in breast cancer due to an alteration in normal epithelial cell polarity caused by an increase in ECM stiffness [150]. Except for hemopoietic cells, all cells need to adhere to a solid surface in order to survive and advance through the cell cycle. Cancer cells are able to invade several organs because they no longer depend on anchoring and cell/surface tension [151]. Metastatic cells require highly controlled biomechanical interactions with their physical surroundings in order to break through the basal lamina, invade blood vessels, evade the bloodstream, and form new tumors [140].

Table 1 describes different disorders arising from deregulated or malfunctioned integrin and integrin-associated protein-mediated mechano-signal transduction.

## 9. Discussion

This review presents comprehensive roles of integrins and integrin-associated proteins in mechano-signal transduction. It presents a comprehensive mechanism of integrin-mediated mechano-signal transduction along with the possible pathologies resulting from deregulated mechano-signal transduction. Integrins are receptor proteins that, during mechano-signal transduction, sense the extracellular mechanical changes [21]. The extracellular mechanical changes serve as mechanical cues for the cell, and integrins perceive them in the form of mechanical signals leading to the binding of integrins to ECM ligands [157]. Upon binding with ECM ligands, integrins get activated and start to cluster [158]. The cytoplasmic tails of β-integrin recruit and activate a number of mechano-sensitive proteins that contribute to FA. Being involved in both integrin-mediated outside–in and inside–out, FA serves a bidirectional role in integrin-mediated mechano-transduction [6,159]. Furthermore, FA is a hub of different downstream signaling pathways, which produce cells’ ultimate response to the extracellular mechanical signal [2]. Thus, integrin-mediated FA is very crucial in integrin-mediated mechano-signal transduction.

Mechano-signal transduction is vital for the cell as it helps in the maintenance of homeostasis in accordance with extracellular mechanical cues. However, mechano-signal transduction is a very crucial process for the cell as it is responsible for both normal and abnormal physiology of the cell. It has been reported that prolonged and extreme mechanical stimulation becomes pathological and results in different diseases, including fibrosis, tumorigenesis, and resistance to cancer immunotherapy [2]. Thus mechano-signal transduction also plays a critical role in disease progression.

Due to the involvement of integrin-mediated mechano-signal transduction in different diseases, the key proteins involved in integrin mechano-transduction serve as potential targets for therapy development. Many drugs targeting the key regulators of mechano-transduction have been investigated for the treatment of diseases arising due to mechano-signal transduction [2]. Many drugs have been tested pre-clinically while others are in different phases of clinical trials. These drugs targeting the key regulators of integrin-mediated mechano-transduction include inhibitors of FAK, c-Src, ILK, and integrin. In this regard, Defactinib is a FAK inhibitor, which is under clinical trial to treat ovarian cancer [160]. Similarly, Dasatinib is a clinically approved c-Src inhibitor that is administered against chronic myeloid leukemia [161]. QLT0267 is a drug that targets ILK and is pre-clinically tested against breast cancer [162]. Eptifibatide is an integrin inhibitor that has undergone clinical trials to treat thrombosis [163]. Thus, targeting the proteins involved in integrin-mediated mechano-signal transduction has the potential to develop therapy against different diseases arising from mechano-transduction.

The extensive research on integrin and its associated protein-mediated mechano-transduction is a promising field. It provides the footprint for the development of effective therapies against different diseases in the future. Moreover, in the future, further inhibitors against different proteins of mechano-signal transduction can be developed. However, there are certain obstacles and challenges to this aspect, which potentially include drug resistance [164]. Similarly, the cytotoxicity of therapy is another aspect that poses a challenge to the therapy development [165]. These challenges propose the need for further elaborate and deep research on integrin-mediated mechano-transduction and experimental techniques for therapy development.

## 10. Conclusions

In conclusion, integrins are mechano-sensitive cell surface receptors that, upon sensing mechanical cues, become involved in mechano-signal transduction. It is responsible for modulating cell proliferation, survival, and migration. Mechano-signal transduction is very crucial for the cell as it is involved in both normal and diseased conditions. Being involved in different diseases, integrins and their associated proteins serve as potent therapeutic targets. Many drugs that are inhibitors of integrins, and integrin-associated proteins are under pre-clinical and clinical trials for treating different disorders involving aberrant mechano-transduction due to abnormal extracellular mechanical cues. There are challenges to integrin-mediated mechano-signal transduction-targeted therapy, including drug cytotoxicity and resistance, which need to be resolved. Further extensive deep research to develop more drugs, targeting mechano-transduction, will be a promising step in the field of therapeutics.

## Figures and Tables

**Table 1:** Different disorders arising due to different proteins involved in integrins and their associated proteins-mediated mechano-signal transduction.

Disorder	Characteristic Symptoms	Key Regulators Responsible for the Progression	References
Atherosclerosis	Blood vessels with plaque deposition, inflammation, and high risks of stroke	α5β1 integrins in response to mechanical force due to blood flow activate FAK and Src, which in turn, via downstream signaling, facilitate the expression of genes that promote plaque deposition.	[152]
Pulmonary fibrosis	Stiffness and scarring of lungs	In response to ECM stiffness, integrins αvβ6 stimulate TGFβ1, mediating YAP/TAZ to increase the expression of genes of fibrosis.	[153]
Pulmonary arterial hypertension	Chest and breath discomfort and heart arrest	In response to mechanical stress on the pulmonary artery, integrin αvβ3 activates Src, which in turn triggers the YAP/TAZ pathway leading to the expression of genes involved in ECM remodeling, which in turn contributes to pulmonary hypertension.	[137]
Alzheimer’s Disease	Neurodegeneration leading to memory shortness	αvβ5 integrin response to Aβ-amyloid accumulation via FAK and ILK, which causes neuro-disruption due to the presence of Aβ amyloid.	[154]
Rheumatoid arthritis	Inflammation and deformation of joints due to autoimmunity	Integrin β2 and β7 mediate the immune cell migration to the inflamed joints. Furthermore, integrins activate NF-κB and JNK pathways, which result in the expression and release of cytokines that further trigger the inflammation and deformation of joints.	[43]
Epidermolysis bullosa	The fragility of the skin leading to blistering on minor forces	In keratinocytes, disruption of interactions between integrin α6β5 and laminin at the basal membrane causes the fragility of the skin, leading to blistering on the little shear.	[155]
Duchenne Muscular Dystrophy	Muscular weakness with difficulty in walking and respiratory issues	α7β1 integrins fail to mediate the attachment of muscle cells with ECM due to the failure of talin and filamin to attach the actin element of the cytoskeleton with integrins, which leads to muscle deformation.	[156]

## Data Availability

Not applicable.
